# The role of real-world data in the development of treatment guidelines: a case study on guideline developers’ opinions about using observational data on antibiotic prescribing in primary care

**DOI:** 10.1186/s12913-019-4787-5

**Published:** 2019-12-05

**Authors:** Stephanie Steels, Tjeerd Pieter van Staa

**Affiliations:** 1 Health e-Research Centre, Farr Institute, School of Health Sciences, Faculty of Biology, Medicine and Health, the University of Manchester, Oxford Road, Manchester, M13 9PL UK; 2 Utrecht Institute for Pharmaceutical Sciences, Utrecht University, Utrecht, the Netherlands

**Keywords:** Clinical practice guidelines, development process, Antibiotic prescribing, Evidence-based medicine

## Abstract

**Background:**

Antimicrobial resistance (AMR) is a prominent threat to public health. Although many guidelines have been developed over the years to tackle this issue, their impact on health care practice varies. Guidelines are often based on evidence from clinical trials, but these have limitations, particularly in the breadth and generalisability of the evidence and evaluation of the guidelines’ uptake. The aim of this study was to investigate how national and local guidelines for managing common infections are developed and explore guideline committee members’ opinions about using real-world observational evidence in the guideline development process.

**Methods:**

Six semi-structured interviews were completed with participants who had contributed to the development or adjustment of national or local guidelines on antimicrobial prescribing over the past 5 years (from the English National Institute for Health and Care Excellence (NICE)). Interviews were audio recorded and transcribed verbatim. Data was analysed thematically. This also included review of policy documents including guidelines, reports and minutes of guideline development group meetings that were available to the public.

**Results:**

Three key themes emerged through our analysis: perception versus actual guideline development process, using other types of evidence in the guideline development process, and guidelines are not enough to change antibiotic prescribing behaviour. In addition, our study was able to provide some insight between the documented and actual guideline development process within NICE, as well as how local guidelines are developed, including differences in types of evidence used.

**Conclusions:**

This case study indicates that there is the potential for a wider range of evidence to be included as part of the guideline development process at both the national and local levels. There was a general agreement that the inclusion of observational data would be appropriate in enhancing the guideline development process, as well providing a potential solution for monitoring guideline use in clinical practice, and improving the implementation of treatment guidelines in primary care.

## Background

Evidence-based medicine (EBM) has been one of the defining features of health care policy and practice since the 1990s. The evidence base for clinical decision-making is built upon critical assessment of relevant literature and assessment of the strength of scientific evidence to inform clinical practice along with clinical reasoning [1, 2]. Clinical practice guidelines, hereafter referred to as guidelines, have formed a cornerstone of the translation of EBM into practice. The route from scientific literature to a new or updated guideline passes through the development of assessments of evidence by a group of guideline committee members and experts in a medical topic. This process of development of guidelines is not straightforward and has been characterised as a ‘black box’ [2]. In developing guidelines, other decisive factors have been identified that play a role alongside the ‘robustness’ of scientific evidence, such as reasoning based on practical or political repertoires [3] and views from members of guideline development groups [2]. The guideline committee members who develop and adapt a guideline, thus develop and adapt guidelines based on a variety of sources, over and above the available evidence. This differs to EBM where other types of studies, such as cohort and case-control studies may also be considered [1].

Since 1999, National Institute for Health and Care Excellence (NICE) role is to improve outcomes for people using the National Health Service (NHS) and other public health and social care services. NICE do this through: producing evidence-based guidance and advice for health, public health and social care practitioners; developing quality standards and performance metrics for those providing and commissioning health, public health and social care services; and, providing a range of information services for commissioners, practitioners and managers across the spectrum of health and social care [4].

Although many guidelines have been developed over the years, their impact on health care practice varies [5]. The acceptance [6] and particularly the implementation of guidelines by doctors, other health care practitioners, and administrators often remain low [7, 8]. Many reasons for guidelines not being adopted and implemented have been brought forward. These include social and political processes, factors relating to health care professionals, factors relating to patients, and factors relating to the guideline itself [6, 9]. To bring best evidence into best practice, improvements in each of these domains are necessary [8]. Guideline development and adaptation in particular appear to be important intervention points for improving the implementability of guidelines [10].

The usability and implementability of guidelines can be improved by broadening the evidence base, and by bringing the evidence that is used and clinical practice closer together [11]. In identifying evidence to inform the development and adaptation of guidelines, preference is often given to randomized clinical trial (RCT) data. RCTs give valuable insight into the effectiveness of medications and other treatments. However, patient populations in these studies are often small with limited follow-up, and as RCTs are often costly to conduct, they cannot always be reproduced in all local circumstances. Moreover, by definition the patients included in RCTs conform to multiple inclusion criteria. In many cases there is little evidence on a sizeable group of patients who do not conform to the inclusion criteria of the studies used, or patients in various countries and settings [12]. Furthermore, evidence from RCTs is often summarized in systematic reviews, which may make the presence or absence of diversity in the included patient populations less apparent. In clinical practice, patient populations are far more diverse than participants in RCTs, which complicates the task of finding applicable and rigorous evidence [13]. The use of a broader evidence base in guideline development and adaptation has therefore been advocated to reduce the gap between evidence and practice.

Use of evidence that identifies a patient’s risk of developing a disease is highly relevant for identifying patient groups that need to be targeted with treatment. This type of data analysis, however, cannot be captured in clinical trial-derived evidence, as this needs incorporation of data about variability within a population. In guidelines on the prevention of cardiovascular events, evidence on identifying patients’ risk of a cardiovascular event has been incorporated [14, 15]. However, other guidelines to date have not made use of such types of evidence [16]. One of the factors contributing to this may be that standards for assessing the quality of risk data analytics have not been developed to the same scientific rigour as standards for incorporating more traditional types of evidence [10]. Therefore, interpretation of this evidence is often left up to the interpretation of individual guideline development group members and group processes [2], which appears to lead few groups to use this evidence. However, incorporating risk based analyses is particularly necessary in guidelines aimed at types of health care that are not specific to one diagnosis and that require complex decisions by health care practitioners [13].

### Relevance of guidelines in prescribing practices in primary care

In primary care, the prescription of antibiotics leads to discomfort in decision-making [17], due to a large degree of diagnostic uncertainty [18]. Such decisions, moreover, occur frequently in primary care practices, as patients often attend their General Practitioner (GP) with symptoms such as acute sore throat [19] or acute cough [20, 21], for which antibiotic treatment is one of the available options. Previous studies have shown that antibiotic treatment for acute sore throat is effective when a bacterial infection is present [22]. However, patients with a low risk of complications from the infection are likely to gain only a small benefit from taking antibiotics. At the same time, rising concerns about antimicrobial resistance lead to the need to diminish antibiotic use [23]. Antibiotic prescribing, then, needs to be targeted foremost at patient groups who are most in need of them, that is, patients who are at high risk of developing infection-related complications.

Prescribing behaviours appear to be based on symptoms rather than on an assessment of the risks to the patient, as the latter is often unclear to GPs [19]. Incorporating evidence about the risks to patients of complications relating to an infection, or relating to the use of common treatments such as antibiotics could facilitate and improve decision-making in primary care [20, 21]. At present, moreover, it is unclear whether antibiotic prescribing decisions are based on the use of guidelines [18]. More intelligent implementation strategies for antibiotic prescribing guidelines have been called for [24]. Thus, there is a need for bringing more clinically relevant evidence into guidelines to improve their uptake by GPs.

The aim of this study was to investigate how national and local guidelines for managing common infections are developed and explore guideline committee members’ opinions about using real-world observational evidence in the guideline development process.

## Methods

This case study is based on interviews with guideline developers involved in the creation of national and/or local guidelines for antimicrobial prescribing, as well as on a documentary analysis of existing national and local guideline information. Data were collected between September 2018 and March 2019.

### Data collection

Research team members from the Health E-Research Centre at the University of Manchester conducted six semi-structured face-to-face interviews were conducted with participants who had contributed to the development or adjustment of national or local guidelines on antimicrobial prescribing over the past 5 years. The national guidelines were developed by the NICE in England. Interviews focused on the types of evidence used in the guideline development process and potential challenges in changing prescribing behaviour for treating common infections. An interview guide was developed to explore and capture guideline developers’ experiences and opinions regarding the how evidence was chosen and the challenges of incorporating other forms of evidence within the development process.

The interview guide also included three vignettes that were developed by the research team: the first vignette presented two published research studies. Both studies gave information about two different types of scoring tools for diagnosing sore throat in clinical practice; guidelines recommended the clinical use of these tools [25, 26]. The second vignette presented data from the Clinical Practice Research Datalink (CPRD), which consists of anonymized patient records from over 400 primary care practices in England that were linked to hospital admission records. CPRD data was then compared with antibiotic prescribing data for acute sore throat from a recently published study [25] that compared different antibiotic prescribing strategies in order to explore the generalisability of this study. The third vignette showed three figures based on CPRD data: the first showed the variability of GP antibiotic prescribing for sore throat (or Upper Respiratory Tract Infection (URTI)) since the year 2000; the second showed average prescribing rates for each year with the three main changes in the rate of prescribing indicated in red; and the third figure showed average prescribing rates for each year with yellow lines indicating the changing/updating of guidelines and red lines indicated the three main changes in the rate of prescribing. The third vignette was presented in order to explore the (lack of) changes in primary care antibiotic prescribing despite the introduction of several guidelines.

Interviews lasted between 45 to 90 min. They were audio-recorded and transcribed verbatim, with respondents then sent a copy of the transcript to review and make any necessary amendments. In addition to the interviews, policy documents including guidelines, reports and minutes of guideline development group meetings, were collected from NICE and the Greater Manchester Clinical Standards Board. Where scientific evidence was cited in these guidelines, this was also collected. All information was drawn from publically available data.

### Data analysis

Thematic analysis was used to analyse interview and document data [27]. Thematic analysis is a widely used method to identify, organise, analyse and report patterns or themes within qualitative research data. Themes were compared across participants and documents. Data analysis took place alongside data collection, in order to ensure that emerging themes from the preliminary analysis will sensitize the researcher to important topics during the interviews, and to ensure that an appropriate assessment of data saturation could be made. NVivo 11 (QSR International Pty Ltd., 2014) was used to aid in data management and analysis.

### Ethical considerations

Participation in the study was based on informed consent and was voluntary. All participants received a participant information sheet and were asked to sign a consent form prior to starting the interview. This study received ethics approval from the University of Manchester Research Ethics committee, Manchester, U.K (Ref: 2018–4682-6712).

## Results

Three key themes emerged through our study: 1) *Perception* versus *actual guideline development process, 2) Using other types of evidence in the guideline development process, and 3) Guidelines are not enough for changing antibiotic prescribing behaviour.* Quotes from participants are used to exemplify and clarify themes. Due to under-recruitment to the study and to further ensure participant anonymity, no further information regarding participant characteristics will be given.

### Theme 1: perception versus actual guideline development process

Whilst publicly available documents gave a general overview of the process involved for developing national guidelines, the interviews provided a more detailed description of the methods used and who was involved throughout the process. In particular, a difference was noted in some areas of the guideline development process, with the majority of the work undertaken by staff at NICE and limited input from committee members and other stakeholders. Several participants described how suggestions made to NICE, such as including expert opinions and a wider range of evidence were considered but not acted upon, preferring to focus efforts on the literature. Whilst all participants reflected that they understood why this was the process, several felt that there was a lot of value in speaking with experts with extensive clinical knowledge and practice.

*“They [NICE] are just concentrating on systematic reviews. And, especially with something like this, the antibiotic guidance, which is so broad, there’s a huge quantity of experience out there that they could ask about for each of the different guidance. I’ve said each time, for each one, they ought to get other people involved.”*

The guideline development process for developing guidelines at the local level was not well documented, with documentation published online for the year 2017 to 2018 only. As a result, interviews with participants who contributed to local guideline development provided useful insight in this area. Participants were able to describe a clear difference between national and local guideline development. Locally, an emphasis was placed on ensuring that local contexts and issues were built into the guidelines whilst cross-referencing national guidelines. Participants involved in the development of local guidelines discussed how local practitioners were encouraged to raise issues they had encountered when treating patients to ensure that these could be addressed in updated guidelines to ensure practitioners felt confident in their practice. As a result, guidelines are updated to reflect these issues and any other localised/national changes every quarter.

### Theme 2: using other types of evidence in the guideline development process

All participants felt that a much wider range of evidence should be used in national guideline development, rather than relying on systematic literature reviews and RCTs that met strict criteria as set out by NICE. Several participants commented that some of the studies reviewed in the national guideline process were difficult to generalise to the U.K population because they were either a systematic literature review or the RCT was conducted in a very specific type of population or in a different country.

Whilst some participants were able to reflect as to the rational for why NICE restrict their studies to systematic literature reviews and RCTs, all participants noted that other studies that provided evidence for best antibiotic prescribing behaviour using other research methods were being excluded (such as qualitative research and observational studies). Many felt that given the current urgency to address antibiotic resistance, there was the potential that other types of studies, such as a qualitative study, could provide useful insight into prescribing behaviours that could then be considered in guideline development.

*“They have a very strict criteria about what guidance they use, and it’s on a hierarchical basis. So, they, basically, will use a systematic review, if that’s available of the area, and then if there aren’t any systematic reviews they will go to RCTs. But, they would not go to RCTs if there was a good systematic review. And then, they really very rarely go anywhere else for any other information. I think they should use qualitative evidence, as well, because I think that’s really useful and it gives you more valuable insight into how things work in real life, which you don’t get with a RCT or systematic review.”*

The interview included asking participants a series of questions based on three vignettes. Vignette one presented two published research studies. Both studies gave information about two different types of scoring tools for sore throat. Nearly all participants were familiar with the scoring tools presented which prompted discussions around how such scoring tools can be useful in clinical practice to support decision making. Despite the limitations of the first scoring tool, the majority of participants felt that having some form of tool to use in clinical practice was better than having none at all. Some participants who were familiar with the second study noted that whilst this was a more appropriate study to consider as it was conducted in the U.K compared to the first scoring tool that was developed in the U.S; this study did not meet the NICE requirements for inclusion in the guideline development process. Despite its limitations, some felt it was a missed opportunity for a recently U.K developed scoring tool to be included in the NICE guideline development process.

Moving through the first vignette, participants tended to focus on the table of information which presented the predictive value of bacterial clinical scoring methods for complication, describing how useful it was to see the data presented for each study side by side. Participants indicated that presenting all results in this manner during the guideline development process would be more useful.

Vignette two presented compared UK primary care CPRD data on antibiotic prescribing for acute sore throat to a recently published study that compared different antibiotic prescribing strategies. Nearly all participants noted how interesting it was to see the research study data presented side by side with population data. In particular, being able to see the study data with the corresponding CPRD data made participants feel more confident in interpreting the results of published studies, as well as providing additional evidence that could assist in determining whether specific interventions could be generalised to the U.K population. All participants felt that having study results presented alongside the relevant population dataset would be beneficial in future guideline development.

*“It’s really interesting to see the study compared with the population. Reading the study, I felt it was as representative as you could get in general practice, but seeing it here with CPRD data is very beneficial because RCTs tend to have a particular type of population which might not represent Wigan. It just makes you feel a bit more confident in the published stuff.”*

In discussing the third vignette, most participants found the third figure within the vignette to be the most useful. The third figure shows average prescribing rates for each year with yellow lines indicating the changing/updating of guidelines and red lines indicated the three main changes in the rate of prescribing. Participants noted how the combination of information was complementary, with several suggesting the addition of key flu outbreaks (such as Swine Flu) labelled would make this figure even more valuable, particularly in monitoring antibiotic prescribing over time using local data.

*“Things like if you’re looking at something like antimicrobial prescribing, it would be useful to see when there were bad years for flu, like swine flu…when those happened and what the knock-on impact of that was, you know, other outbreak kind of related stuff. When there were…I don’t know whether…if there’s a really bad winter, does that make a bigger impact on antibiotic use in terms of more transmission of…you know, is there more transmission of upper respiratory tract infections, things like that. I know obviously the flu does have an impact, so that would be useful to see in the data.”*

### Theme 3: guidelines are not enough for changing antibiotic prescribing behaviour

Opinions about the usefulness and effectiveness of national guidelines for antibiotic prescribing varied. All participants stated that they were aware of current governmental policies and guidelines aimed at reducing antibiotic prescribing. However, participants involved in some form of clinical practice admitted to rarely using national guidelines in practice, relying more on the expertise of other clinical professionals, their own clinical knowledge or a combination of locally produced guidelines and expert knowledge. Most participants stated there were other guidelines for antibiotic prescribing that were more useful to prescribing practitioners on a day-to-day basis. Participants commonly referred to the guidelines produced by Public Health England and locally produced guidelines when asked what other types of guidelines either they themselves used or their colleagues used in practice. In describing what they felt was more useful about these other guidelines; participants noted the usefulness of having information presented in a tabulated format on the different types of antibiotics for a range of health conditions.

All participants noted that changes and updates to NICE antibiotic prescribing does not necessarily result in reductions in antibiotic prescribing. There was a mixed response as to whether GP’s were monitored on their prescribing behaviour. Regional variations were noted in how antibiotic prescribing was monitored. It was noted that some local Clinical Commissioning Groups (CCGs that are organisations responsible for the planning and commissioning of health care in a region) were more proactive than others, whilst other CCGs did not have the capacity to provide additional support to GPs in reducing prescribing behaviours. Financial incentives were deemed not to work as they were too negligible to make a difference to prescribing behaviours, particularly those who work in high prescribing areas. One suggestion was to have localised targets that were attainable, rather than a national target.

*“They’re quite strange targets really because they’re arbitrary, and based on an England mean from 2013. It doesn’t account for anything that’s issued from walk-in centres, out-of-hours places that use different budgets and private prescriptions, which in affluent areas, GPs might offer both. So if you’re not going to make it, it’s not an incentive to reduce your prescribing behaviour. Plus there’s no penalty and the financial incentive isn’t worth it if you’re already struggling to meet that target. What we should do is have more localised targets that address the contexts we work in…that way if becomes more like comparing apples with apples, rather than apples with pears.”*

Another issue that was raised by participants was that national guidelines do not take into account the localised contexts, such as areas with a higher rate of elderly patients, which often results in higher rates of prescribing. Many felt that as a result of this, many GPs and other forms of prescribers simply did not bother to look at the NICE guidelines, preferring to use more localised versions that provided recommendations specific to the populations that they worked with. In addition, several participants highlighted that prescribing data did not necessarily represent prescribing behaviour, particularly if GPs offer private prescriptions.

Several participants reflected on the pressures that GPs face in seeing a patient within a 10-min appointment slot. It was suggested that providing GPs with additional support (such as mentoring), regular meetings to discuss difficult cases and learning from others in how they have able to tackle these types of cases, as well as a forum to discuss guidelines/new regulations, were key to reducing antibiotic prescribing in GPs. At the same time, participants who discussed these issues also noted the importance of sharing the responsibility with the public, rather than it being the sole responsibility of the GP or healthcare practitioner. Suggestions were made for the Government to provide more public health education campaigns to the general public, with targeted approaches to population groups who may insist on being given antibiotics when there is not a medical need.

## Discussion

Although many guidelines have been developed over the years, particularly on managing common infections, their impact on health care practice varies. Treatment guidelines are often based on assessments of evidence from clinical trials or systematic literature reviews, but these have limitations, particularly in representing the whole population of patients in primary care. This suggests that there is a need for bringing more clinically relevant evidence into guidelines to improve their uptake in clinical practice.

This case study provides an in-depth look at the guideline development process within treating common infections, the challenges of reducing antibiotic prescribing that go beyond the guideline process and how other forms of evidence could be utilised in the guideline development process. Three key themes emerged through our study: 1) Perception versus actual guideline development process, 2) Using other types of evidence in the guideline development process, and 3) Guidelines are not enough for changing antibiotic prescribing behaviour. In addition, our study was able to provide some insight between the documented and actual guideline development process within NICE, as well as how local guidelines are developed.

Whilst discussing other types of evidence, such as the availability of population data, all participants stated that they would welcome additional evidence that provides them with relevant population data that could be presented alongside the results of published studies. In addition, interview participants were able to reflect on the constraints imposed onto NICE in the guideline development process by other stakeholders within government.

NICE guidelines make evidence-based recommendations on a wide range of topics, from preventing and managing specific conditions, improving health and managing medicines in different settings, to providing social care to adults and children, and planning broader services and interventions to improve the health of communities [4]. However, implementing these guidelines in practice is not without its challenges when GPs also have to meet other Quality Improvement and Quality Assurance targets.

It has been recommended that in order to be used, clinical guidelines need to be integrated as part of other healthcare quality improvement processes [13, 28]. This will require cooperation between guideline developers and other healthcare stakeholders [13, 29], similar to the types of working that were noted in our study at the local guideline develop level. This strategy has been found to be successful elsewhere. For example, Richter-Sundberg et al. (2015) found that it was possible for the planning and development of guidelines to involved national, regional and local health care providers. Despite the additional time required to utilise such a development process, particularly given the complexities connected to different guideline areas, time and resources available, having a dialogue and making agreements with stakeholders at all levels was found to be valuable.

Our results are based on a single case study of common infection guideline development with an emphasis on antibiotic prescribing. There might be other aspects that need to be considered in other contexts and other ways of achieving reduced levels of antibiotic prescribing in primary care clinical practice. On a more practical level, regular meetings between local and national guideline developers would provide insight into the different patient groups across the U.K and the difficulties of utilising national guidelines in a range of population settings. In addition, further consideration could be given to utilising complementary local data to monitor the effectiveness of local interventions, which could then be considered at the local guideline level.

## Conclusion

This case study has provided insight into the processes for antimicrobial guideline development at the national and local levels in the U.K, particularly in the types of evidence that are currently used. The study has indicated that guideline developers could benefit from including a wider range of published research studies, as well as the input from experts during the early stages of developing or updating antibiotic prescribing guidelines. In addition, the inclusion of population data was found to be a source of evidence that many guideline developers would like to see utilised as part of the wider range of evidence presented to them. However, there will be other aspects that need to be considered in using other forms of evidence within the process of developing guidelines, as well as in how these are used for monitoring purposes. Furthermore, Government and guideline developers will also need to consider focusing on addressing the wider issues that can impact antibiotic prescribing and ensure that the necessary resources and support are put in place for both GPs and the general public to achieve rational antibiotic prescribing.

## Data Availability

Due to confidentiality and anonymity issues caused by the small sample size, we are unable to provide data for other researchers.

## Abbreviations

AMR: Antimicrobial resistance
CCGs: Clinical Commissioning Groups
CPRD: Clinical Practice Research Datalink
EBM: Evidence-based medicine
GP: General Practitioner
NHS: National Health Service
NICE: National Institute for Health and Care Excellence
RCT: Randomized clinical trial
URTI: Upper Respiratory Tract Infection
