# Correction to: The role of real-world data in the development of treatment guidelines: a case study on guideline developers’ opinions about using observational data on antibiotic prescribing in primary care

**DOI:** 10.1186/s12913-020-05335-x

**Published:** 2020-05-26

**Authors:** Stephanie Steels, Marieke Van der Zande, Tjeerd Pieter van Staa

**Affiliations:** 1 Health e-Research Centre, Faculty of Biology, Medicine and Health, Farr Institute, School of Health Sciences, The University of Manchester, Oxford Road, Manchester, M13 9PL UK; 2 Utrecht Institute for Pharmaceutical Sciences, Utrecht University, Utrecht, the Netherlands

**Correction to: BMC Health Serv Res (2019) 19:942**

10.1186/s12913-019-4787-5

Following publication of the original article [1], the author reported that the 2nd author was omitted from the author group. Marieke Van der Zande has been added to the author group and is presented correctly in this correction article. In addition, the content needs to be revised in the Authors’ contributions section.

The updated Authors’ contributions is given below.
